# Customising PRESAGE^®^ for diverse applications

**DOI:** 10.1088/1742-6596/444/1/012029

**Published:** 2013

**Authors:** T Juang, J Newton, M Niebanck, R Benning, J Adamovics, M Oldham

**Affiliations:** 1 Duke University Medical Center, Durham, NC, USA; 2 Rider University, Lawerenceville, NJ, USA

## Abstract

PRESAGE^®^ is a solid radiochromic dosimeter consisting of a polyurethane matrix, a triarylmethane leuco dye, and a trihalomethane initiator. Varying the composition and/or relative amounts of these constituents can affect the dose sensitivity, post-irradiation stability, and physical properties of the dosimeter. This allows customisation of PRESAGE^®^ to meet application-specific requirements, such as low sensitivity for high dose applications, stability for remote dosimetry, optical clearing for reusability, and tissue-like elasticity for deformable dosimetry. This study evaluates five hard, non-deformable PRESAGE^®^ formulations and six deformable PRESAGE^®^ formulations and characterizes them for dose sensitivity and stability. Results demonstrated sensitivities in the range of 0.0029 – 0.0467 ΔOD/(Gy·cm) for hard formulations and 0.0003 – 0.0056 ΔOD/(Gy·cm) for deformable formulations. Exceptional stability was seen in both standard and low sensitivity non-deformable formulations, with promising applications for remote dosimetry. Deformable formulations exhibited potential for reusability with strong post-irradiation optical clearing. Tensile compression testing of the deformable formulations showed elastic response consistent with soft tissues, with further testing required for direct comparison. These results demonstrate that PRESAGE^®^ dosimeters have the flexibility to be adapted for a wide spectrum of clinical applications.

## 1. Introduction

PRESAGE^®^ is a solid radiochromic dosimeter with the potential for use across a wide range of clinical applications [1–3]. In order to maximize PRESAGE^®^’s usefulness as a 3D dosimetry [4] tool, the dosimeter must be adaptable to meet application-specific dosimetry requirements. Examples of application-specific considerations are listed in table 1.

The components of PRESAGE^®^ are a transparent polyurethane matrix (approximately 90% of the PRESAGE^®^ dosimeter by weight), a triarylmethane leuco dye, and a trihalomethane initiator. Formulations customized to specific applications can be made by varying the composition and/or relative amounts of these constituents. Physical characteristics (transparency, elasticity) of the PRESAGE^®^ polyurethane matrix are dependent on the starting monomeric isocyanate and polyol, which are catalyzed by metal compounds. Recent studies have shown that the composition of these metal compounds and the trihalomethane initiator both influence dosimeter sensitivity [5, 6].

This study evaluates several formulations of PRESAGE^®^, including novel deformable formulations under initial stages of development, and characterizes them for a range of dose sensitivities and post-irradiation stability.

## 2. Methods

### 2.1. PRESAGE^®^ Chemistry and Synthesis

The five formulations of the standard hard (Shore D Hardness 80) PRESAGE^®^ (including two low sensitivity formulations specifically designed for high-dose applications) and six formulations of the deformable (Shore A Hardness 10–20) PRESAGE^®^ under investigation are listed in detail in table 2. The leuco dye leucomalachite green (LMG) and its derivatives were synthesized by placing 1 equivalent of the corresponding aldedyde N,N-dimethylaniline (6 eq) and benzene (0.5M with respect to the aldehyde) in a flask fitted with a Dean-Stark trap and reflux condenser. p-Toluenesulfonic acid (0.1 eq) was added to the mixture and the solution refluxed. The reaction was monitored by thin layer chromatography until completion and worked up by diluting with benzene and washing with 10% sodium bicarbonate solution. The benzene and excess aniline were removed via azeotrope distillation with water. Flash column chromatography was performed to further purify the products. One LMG derivative (o-MeO-LMG DEA) was synthesized using N,N-diethylaniline.

### 2.2. PRESAGE^®^ Formulation Characterization

#### 2.2.1. Sensitivity

Sensitivity was determined from the change in optical density in sets of cuvettes containing each formulation under evaluation. Each set of cuvettes was irradiated on a 6 MV linac to known doses between 0 – 25 Gy for low sensitivity formulations and 0 – 8 Gy for all other formulations. Change in optical density was acquired using a Thermo Spectronic Genesys 20 spectrophotometer, and determined by subtracting the pre-irradiation absorption from the post-irradiation absorption for each cuvette at the peak absorption wavelength for each formulation. Cuvettes were stored in the dark following irradiation and tracked for up to 14 days to determine post-irradiation stability.

#### 2.2.2. Tensile testing

The six deformable PRESAGE^®^ formulations were subjected to tensile compression testing to evaluate the elasticity of the formulations. While Young’s modulus values for elastic materials, such as soft biological tissues and deformable PRESAGE^®^, are highly dependent on the method of deformation [1], consistent tensile measurements allow an initial elasticity comparison between formulations. 10 mm × 10 mm × 25 mm samples of each formulation were subjected to compression by 80% (8 mm) at a rate of 2 mm/min using a Lloyd Model LRX Plus tensile tester.

## 3. Results

### 3.1. Sensitivity

The immediate post-irradiation sensitivities of each formulation are listed in table 3. All formulations exhibited a linear dose response. In the non-deformable formulations, use of o-MeO-LMG in the SS3 formulation increased the dosimeter dose sensitivity by approximately two-fold even with decreased initiator content. Deformable PRESAGE^®^ formulations were found to exhibit sensitivities one order of magnitude lower than that of comparable formulations using the non-elastic polyurethane matrix.

### 3.2. Post-irradiation Stability

**Figure 1:**
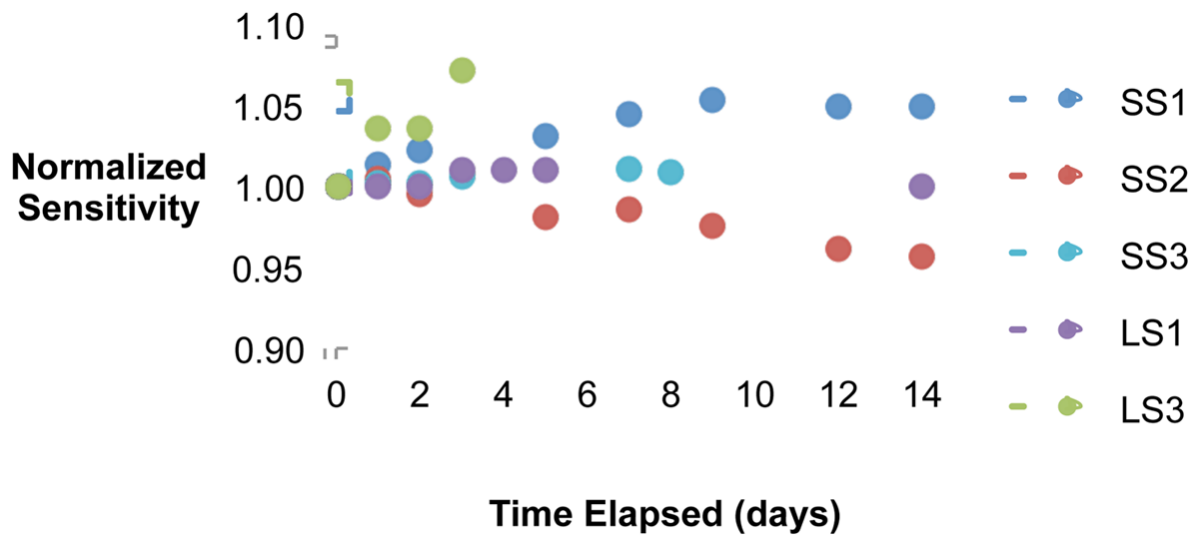
Normalized sensitivity over 14 days for all non-deformable formulations.

Normalized post-irradiation sensitivities over time are shown in figure 1 for the five non-deformable PRESAGE^®^ formulations. This figure illustrates the wide variety of response – from OD darkening (increasing sensitivity over time) to optical clearing (decreasing sensitivity over time) – achievable through modifications to the leuco dye and initiator. Exceptional post-irradiation stability (variation ≤1%) was exhibited by both standard sensitivity (SS3) and low sensitivity (LS1) formulations, suggesting promising applications in remote dosimetry for both standard and high dose applications.

Sensitivity measurements were taken of the two most sensitive deformable formulations (D3 and D4) 2 days following irradiation as an initial assessment of post-irradiation characteristics (table 3). Both formulations exhibited decreases in sensitivity and ΔOD, suggesting potential reusability.

### 3.3. Elasticity of Deformable Formulations

**Figure 2:**
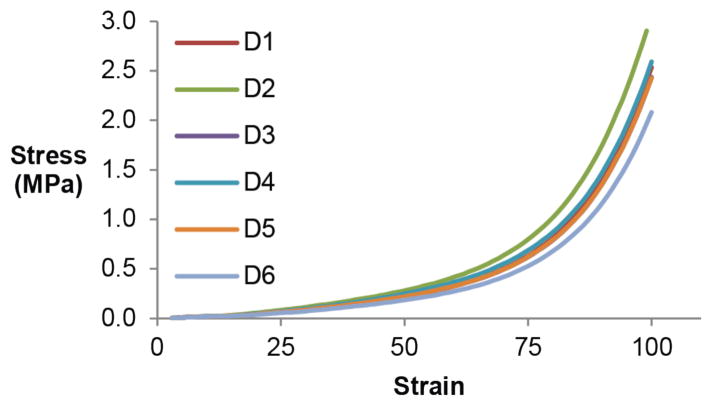
Stress-strain curves for deformable formulations determined through tensile compression. All samples were compressed by 80% (8 mm deflection). Young’s modulus is represented by the slopes of the curves and is stress-dependent.

Stress-strain curves for all deformable formulations are plotted in figure 2. The elastic response of all formulations show a stress-dependent Young’s modulus (stress/strain) and increasing resistance to deformation with increasing applied stress, which is consistent with most soft biological tissues [7]. Further tensile testing is required to directly compare deformable PRESAGE^®^ elasticity to that of tissues of interest.

## 4. Conclusion

All PRESAGE^®^ dosimeter formulations have in common a polyurethane matrix, a triarylmethane leuco dye, and a trihalomethane initiator which can be varied to adjust the properties of the dosimeter to suit application-specific dosimetry requirements. The most frequently used leuco dye has been LMG, but as shown above, formulations synthesized with the LMG derivative o-MeO-LMG are both more dose-sensitive per weight and more stable post-irradiation. Our results demonstrate that PRESAGE^®^ dosimeters have the flexibility to be adapted for use in a wide spectrum of clinical applications, including low sensitivity for high dose applications, elasticity for deformable dose tracking, post-irradiation stability for remote dosimetry, and clearing for reusability.

## Figures and Tables

**Table 1:** Examples of Application-Specific Dosimetry Considerations

Application	Considerations
IMRT, VMAT	Tissue equivalent electron density Volume-independent dose response Un-irradiated transparency
Radiosurgery	High doses require low sensitivity
Remote dosimetry	Post-irradiation stability, low thermal effects
Brachytherapy	Tissue equivalence, high sensitivity, good stability
Deformable dosimetry	Elastic properties comparable to human tissues
Reusable dosimeters	Stable immediately post-RT, then optical clearing

**Table 2:** PRESAGE^®^ formulations under evaluation.

	Formulation	Leuco Dye	Initiator Content (%)	Peak λ (nm)
**Non-Deformable**	SS1 (standard sensitivity)	2.0% LMG	0.75%	633
	SS2 (standard sensitivity)	2.0% LMG	0.50%	633
	SS3 (standard sensitivity)	1.7% o-MeO-LMG	0.40%	633
	LS1 (low sensitivity)	1.5% LMG	0.75%	633
	LS3 (low sensitivity)	1.0% o-MeO-LMG	0.75%	633

**Deformable**	D1	2.0% o-MeO-LMG	0.50%	633
	D2	2.0% o-MeO-LMG-DEA	0.50%	633
	D3	2.0% p-MeO-LMG	0.50%	620
	D4	2.0% dimethyl-LMG	0.50%	633
	D5	2.0% o-Cl-LMG	0.50%	647
	D6	2.0% o-Br-LMG	0.50%	639

**Table 3:** PRESAGE^®^ sensitivity.

	Formulation	Sensitivity (ΔOD/(Gy·cm))		Formulation	Sensitivity (ΔOD/(Gy·cm))	
		Immediate			Immediate	2 days
**Non-Deformable**	SS1	0.0225	**Deformable**	D1	0.0012	--
	SS2	0.0213		D2	0.0014	--
	SS3	0.0467		D3	0.0056	0.0002
	LS1	0.0102		D4	0.0034	0.0028
	LS3	0.0029		D5 D6	0.0005 0.0003	-- --
